# Mechanisms for Host Immune Evasion Mediated by *Plasmodium falciparum*-Infected Erythrocyte Surface Antigens

**DOI:** 10.3389/fimmu.2022.901864

**Published:** 2022-06-15

**Authors:** Akihito Sakoguchi, Hisashi Arase

**Affiliations:** ^1^ Department of Molecular Protozoology, Research Institute for Microbial Diseases, Osaka University, Suita, Japan; ^2^ Department of Immunochemistry, Research Institute for Microbial Diseases, Osaka University, Suita, Japan; ^3^ Laboratory of Immunochemistry, WPI Immunology Frontier Research Center, Osaka University, Suita, Japan; ^4^ Center for Infectious Disease Education and Research, Osaka University, Suita, Japan

**Keywords:** *Plasmodium falciparum*, immune evasion, variant surface antigens, RIFIN, inhibitory immune receptors, receptor-containing antibodies

## Abstract

*Plasmodium falciparum* infection causes the most severe form of malaria. It has been hypothesized that *P. falciparum* directly suppresses host immune responses because sufficient acquired immunity is often not induced even by repeated *P. falciparum* infections in malaria-endemic areas. It is known that many kinds of *P. falciparum*-derived proteins are expressed on the surface of *P. falciparum*-infected erythrocytes (IEs), and these proteins have long been thought to be a key to the elucidation of the host immune evasion mechanisms. Our recent studies have revealed that the *P. falciparum*-derived erythrocyte surface antigen, RIFIN, the largest multiple gene family protein in the *P. falciparum* genome, suppresses host immune cell activation through direct interaction with human inhibitory immune receptors. In this review, we will discuss the molecular mechanisms for host immune evasion by *P. falciparum*-infected erythrocyte surface antigens. In addition, we will discuss the recently identified host immune response to *P. falciparum* using specialized antibodies that target host-*P. falciparum*-derived molecule interactions.

## Introduction

Malaria is one of the most important infectious diseases in the world and still affects more than 200 million people annually, resulting in more than 600,000 deaths in 2021 (1). About 80% of deaths are among children under five years of age. Malaria is an infectious disease caused by *Plasmodium* species, of which malaria caused by *P. falciparum* is particularly severe, and most of the deaths are associated with *P. falciparum* infection. *P. falciparum* is transmitted by female *Anopheles* mosquitoes, and sporozoites accumulated in the salivary glands of the *Anopheles* mosquitoes enter the host body during blood feeding. Sporozoites that invade the blood are firstly taken up by hepatocytes, with repeated division of parasites in hepatocytes forming thousands of merozoites. Merozoites eventually destroy the hepatocytes and are released into the blood (liver stage). Soon after release into the blood, merozoites invade erythrocytes and divide into more than 30 merozoites through the ring, trophozoite, and schizont stages. They are then released into the blood by disrupting the erythrocyte membrane, subsequently invading new erythrocytes to repeat the cycle (blood stage). Therefore, *P. falciparum* multiplies through a complex life cycle within the host and is transmitted by the blood feeding of new *Anopheles* mosquitoes. Recent studies have shown that *P. falciparum* maintains infection and successfully evades host immune responses by producing and utilizing a variety of parasite-derived molecules at each stage in this complex life cycle.

Human immune cells express a variety of inhibitory immune receptors to maintain host immune homeostasis. However, some viruses and cancer cells are known to evade host immunity *via* these inhibitory immune receptors. *P. falciparum* infection induces antibodies against various *P. falciparum*-derived proteins in the host throughout multiple stages. However, these antibodies are not enough to effectively protect *P. falciparum* infection. Therefore, *P. falciparum* seems to possess multiple mechanisms of immune evasion. We have recently discovered novel mechanisms by which *P. falciparum* exploits inhibitory immune receptors to evade host immunity. In this review, we will discuss the host immune evasion mechanisms mediated by blood-stage *P. falciparum* infection.

## *P. falciparum*-Infected Erythrocyte Surface Antigens

*P. falciparum* merozoites invade erythrocytes and mature through the ring, trophozoite, and schizont stages, during which *P. falciparum* produces a large number of parasite-derived proteins. Some of these proteins are transported beyond the parasitophorous vacuole (PV) to the cytoplasm of erythrocytes and are subsequently expressed on the surface of the infected erythrocytes (2–4). These erythrocyte surface antigens are derived from multiple gene family proteins located mainly in the subtelomeric region of *P. falciparum* genome and are referred to as variant surface antigens (VSAs) (5). VSAs are mainly composed of *P. falciparum* erythrocyte membrane protein 1 (PfEMP1), *P. falciparum*-encoded repetitive interspersed families of polypeptide (RIFIN), and subtelomeric variant open reading frame (STEVOR). These proteins are encoded by *var* (~60 genes), *rif* (~150 genes), and *stevor* (~30 genes) genes respectively (5) **Table 1**. PfEMP-1 is a well-studied molecule, but there are very few analyses of RIFIN and STEVOR; therefore, much remains unknown.

**Table 1:** Summary of P. falciparum-infected surface antigens in this Review.

Surface antigens	Genes	Gene numbers	Expression stages	Host receptors	Functions	References
**PfEMP-1**	***var***	**~60**	**Trophozoite, Schizont**	**CD36, ICAM-1, EPCR**	**Sequestration of IEs,** **Rosette formation,** **Immunosuppression**	(5–13)
**STEVOR**	***stevor***	**~150**	**Trophozoite, Schizont,** **Gametocyte, Merozoite,** **Sporozoite**	**Glycophorin C**	**Rosette formation**	(5, 14–18)
**RIFIN**	***rif***	**~30**	**Trophozoite, Schizont,** **Gametocyte, Merozoite,**	**Type A erythrocyte antigen,** **Sialic acid on Glycophorin A,** **LAIR1, LILRB1, LILRB2**	**Rosette formation,** **Immunosuppression**	(5, 19–26)

## Human Inhibitory Immune Receptors

**Figure 1:**
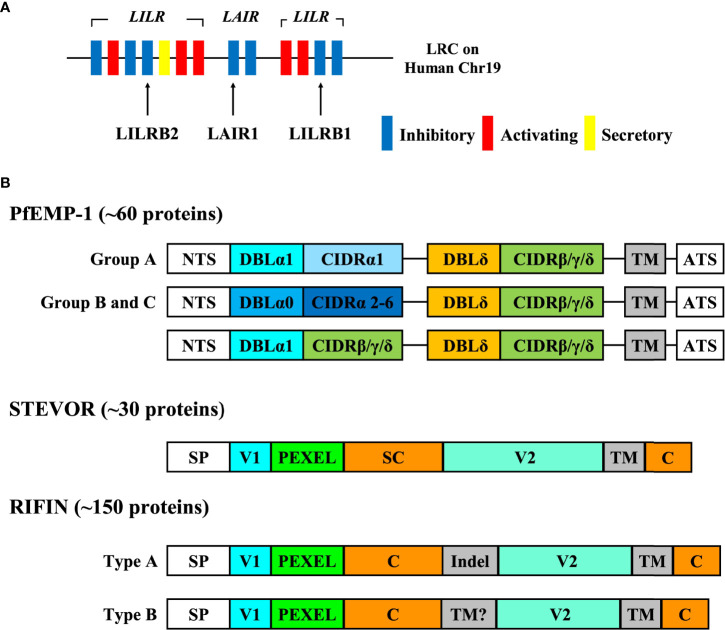
The genomic location of inhibitory immune receptors and the structure of VSAs. **(A)** The genomic location of inhibitory immune receptors. Inhibitory immune receptors are located in the leukocyte receptor complex (LRC) region on human chromosome 19q13.4 in tandem. LRC region contains a number of immunoglobulin-like receptors such as leukocyte immunoglobulin like receptors (LILRs) and leukocyte associated immunoglobulin-like receptors (LAIRs). Each of these receptors forms a multiple gene family, and LILR family consists of five inhibitory receptors (LILRB1-5), five activating receptors (LILRA1, 2, 4-6) and one secretory form (LILRA3). It is thought that activating receptors have evolved from inhibitory receptors to overcome pathogens because some inhibitory receptors such as LILRB1 and LAIR1 are exploited by pathogens in their immune evasion mechanisms.. **(B)** The structure of VSAs. PfEMP-1 proteins are divided into groups (A-C) based on chromosomal location and the direction of transcription. The extracellular region of PfEMP1 is composed of the combination of a Duffy binding-like domain (DBLα-ζ) and a cysteine-rich interdomain region (CIDRα-δ) depending on the organization and the length. This figure shows the typical structure of group (A-C) PfEMP-1. The larger PfEMP1 proteins have additional DBL domains between DBL and CIDR domains. PfEMP-1 can bind to molecules such as CD36, intercellular adhesion molecule-1 (ICAM-1), and endothelial protein C receptor (EPCR) via its CIDR and DBL domains. RIFIN is divided into type A and type B RIFIN, and type A RIFIN contains a 25 amino acid sequence inserted at the N-terminus (Indel) that is not present in type B RIFINs. RIFIN interacts with immune receptors via its variable region (V2).

Human immune cells express a wide variety of inhibitory immune receptors that interact with self-ligands such as MHC class I molecules for appropriate regulation of immune responses. Inhibitory immune receptors have immunoreceptor tyrosine-based inhibitory motifs (ITIMs) in the cytoplasm and transduce inhibitory signals into the immune cells by associating with Src-homology 2-containing tyrosine phosphatase-1 and -2 (SHP-1, SHP-2) (27). A large number of inhibitory immune receptors have been reported previously, and these receptors are mainly composed of two groups, the leukocyte receptor complex (LRC) (28) (**Figure 1A**) and the NK gene complex (NKC) (29).

Some viruses and cancer cells are suggested to evade host immunity *via* these inhibitory immune receptors. For example, mouse cytomegalovirus (MCMV) expresses m157, an MHC class I-like molecule, as a ligand for the mouse inhibitory immune receptor, Ly49I (30). Human cytomegalovirus (HCMV) up-regulates the expression of a non-classical HLA class I molecule HLA-E by the HCMV-derived protein gpUL40 and suppresses NK cell function *via* inhibitory immune receptors, CD94/NKG2A (31). Cancer cells have been reported to inhibit phagocytosis from tumor-associated macrophages *via* MHC class I-LILRB1 signaling (32). Therefore, it has been suggested that inhibitory immune receptors are involved in the immune evasion mechanisms of pathogens and cancer cells. Furthermore, these inhibitory immune receptors have also been suggested to be involved in the immune evasion mechanisms of *P. falciparum* as described below.

## Mechanisms for Host Immune Evasion by *P. falciparum*-Infected Erythrocyte Surface Antigens

### -Antigenic Variation

*P. falciparum*-infected erythrocytes (IEs) express erythrocyte surface antigens referred to as VSAs, but these antigens can be targets of host immune responses, such as the production of antibodies. For example, PfEMP-1 is a major target antigen for antibodies, and antibodies against PfEMP-1 specifically promote the elimination of IEs (33). Therefore, *P. falciparum* transcribes and translates molecules such as PfEMP-1 and STEVOR, multiple gene family proteins, without expressing all the variants of each molecule on the IEs at once. Rather, only a single gene is transcribed and translated in the case of PfEMP-1 (34) and only a few genes in the case of STEVOR (35), with all the other genes being silenced. This phenomenon is termed ‘antigenic variation’ and it allows *P. falciparum* to evade the host immune responses including antibodies by switching the antigen expression patterns on IEs. The molecular mechanism underlying ‘antigenic variation’ has been well characterized in the *var* gene family encoding PfEMP-1, and it is thought to be regulated by an epigenetic mechanism (36).

### -PfEMP-1

PfEMP-1 is a high-molecular weight protein (200-350 kDa), and the extracellular region of PfEMP-1 is composed of cysteine-rich interdomain regions (CIDR) domains and Duffy-binding-like (DBL) domains (6) (**Figure 1B**). PfEMP-1 is known to be primarily expressed on the surface of IEs in the trophozoite and schizont stages. PfEMP-1 promotes the sequestration of IEs into the vessel walls to prevent the destruction of IEs in the spleen by binding to molecules expressed on vascular endothelial cells such as CD36, intercellular adhesion molecule-1 (ICAM-1), and endothelial protein C receptor (EPCR) through its CIDR and DBL domains (7). The IE sequestration into the cerebral microvasculature is known to cause a severe form of malaria, cerebral malaria (CM) (8). It has also been shown that IEs form aggregates, termed ‘rosettes’, by binding to non-infected erythrocytes *via* PfEMP-1 (9). The formation of these rosettes is thought to interfere with recognition by immune cells and antibodies (10). PfEMP-1 has been reported to have direct effects on host immune cells beyond sequestration and rosette formation. Antigenic variation allows IEs to escape from antibody responses by altering the expression patterns of PfEMP-1 (5). The stimulation of PfEMP-1 suppresses NF-κB activity in monocytes and macrophages, and eventually causes the suppression of cytokine and chemokine production (11). Further, PfEMP-1 binds to dendritic cells (antigen-presenting cells) *via* CD36 and CD51, and significantly reduces their antigen-presenting ability (12), while also suppressing cytokine release by innate lymphocytes such as NK cells and γδ T cells (13). Therefore, PfEMP-1 induces direct immunosuppressive effects on various types of immune cells.

### -STEVOR

STEVOR is a small-molecular weight protein (30-40 kDa), with the extracellular region of STEVOR mainly composed of semiconserved and hypervariable regions (14) (**Figure 1B**). STEVOR is expressed in various parasite stages, such as trophozoites, schizonts, gametocytes, merozoites, and sporozoites (15–17), and it has been suggested that STEVOR has different functions in each stage. The function of STEVOR as an IE surface antigen and a merozoite surface antigen is to bind glycophorin C (GPC) on erythrocytes *via* the semiconserved region in STEVOR and form rosettes with uninfected erythrocytes in a PfEMP-1-independent manner (18). Antibodies against STEVOR inhibit merozoite invasion into erythrocytes; therefore, it is suggested that the formation of these rosettes *via* STEVOR promotes merozoite invasion into erythrocytes (18). However, very limited analysis of STEVOR has been performed, and several things remain unknown.

### -RIFIN

RIFIN is a small-molecular weight protein (27-45 kDa) and is the largest multiple gene family protein in *P. falciparum*, encoded by more than 150 *rif* genes in each *P. falciparum* genome (19) (**Figure 1B**). RIFIN is one of the erythrocyte surface antigens expressed on IEs, gametocytes, and merozoites and as in the case of the *stevor* genes, only a few out of more than 150 *rif* genes thought to be transcribed and expressed on the surface of IEs at once (20, 21). RIFINs are divided into type A and type B RIFINs based on their structural specificity. Type A RIFINs, comprising 70% of all RIFINs, contain a 25 amino acid sequence inserted at the N-terminus that is not present in type B RIFINs (19, 22). Irrespective of type, RIFINs are composed of N-terminal and C-terminal conserved regions and variable regions (V2) located between these two conserved regions (**Figure 1B**). It has been reported that the function of a single type A RIFIN of the laboratory strain is to bind to the type A erythrocyte surface antigen and sialic acid on glycophorin A (GPA) for rosette formation (21), but the function of most RIFINs remains unknown. It has been suggested that *P. falciparum* immune evasion mechanisms might exist, similar to those seen in viral infections, as the induction of sufficient acquired immunity is difficult even after repeated infections with *P. falciparum* in malaria-endemic areas. Our recent studies have shown that some RIFINs bind to human inhibitory immune receptors such as LILRB1, LILRB2, and LAIR1 (23, 24). We will discuss the characteristics of each receptor-binding RIFIN in the following sections.

### -LILRB1-Binding RIFIN

**Figure 2:**
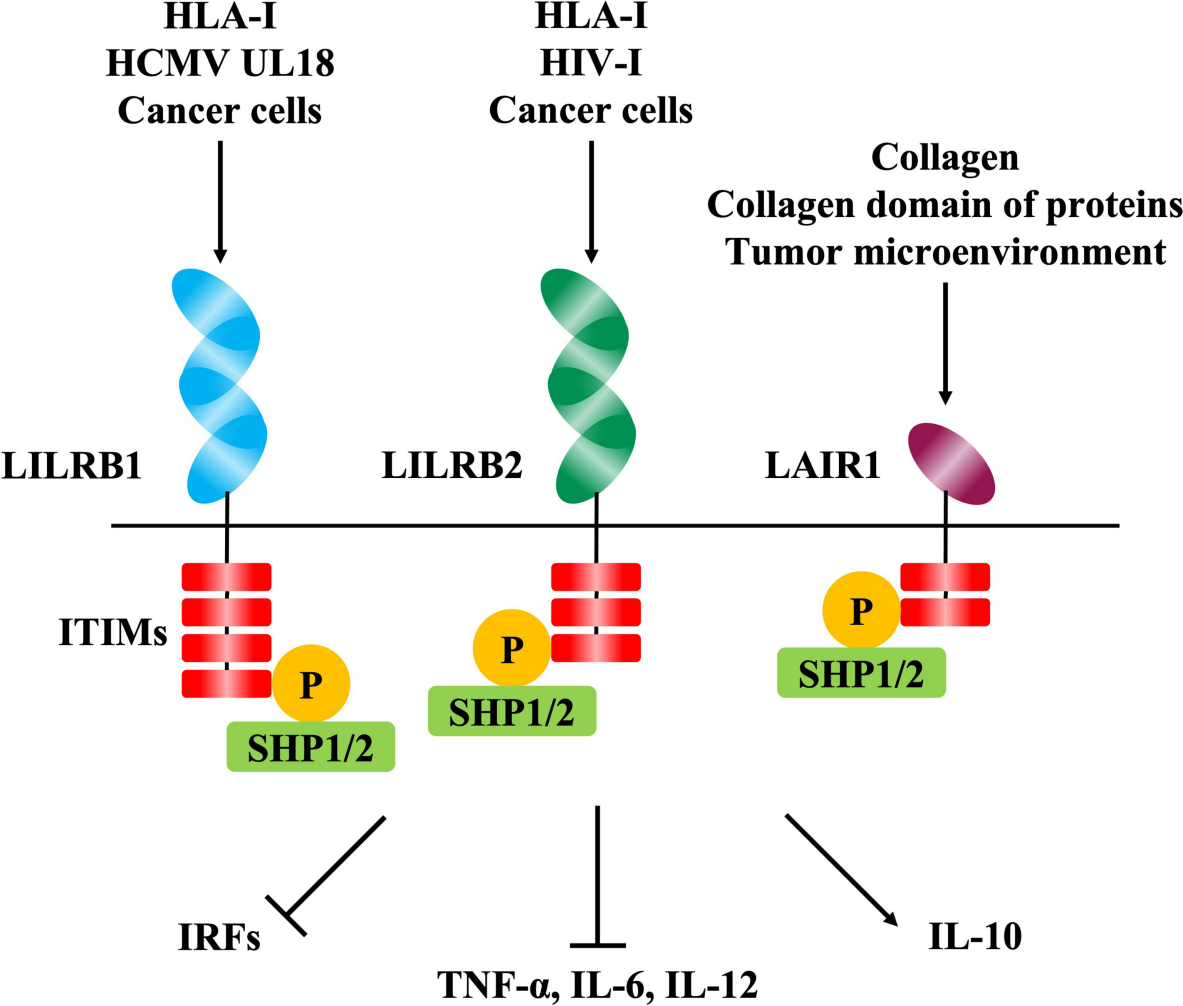
The signaling pathways mediated by inhibitory immune receptors. The phosphorylation of immunoreceptor tyrosine-based inhibition motifs (ITIMs) in the intracellular domain is induced by the binding of ligands to the extracellular domain of inhibitory immune receptors including LILRB1, LILRB2, and LAIR1. Phosphorylation of ITIMs induces phosphatases such as SHP-1 and SHP-2, resulting in immunosuppressive effects such as decreased cytokine production *via* various intracellular signaling pathways.

**Figure 3:**
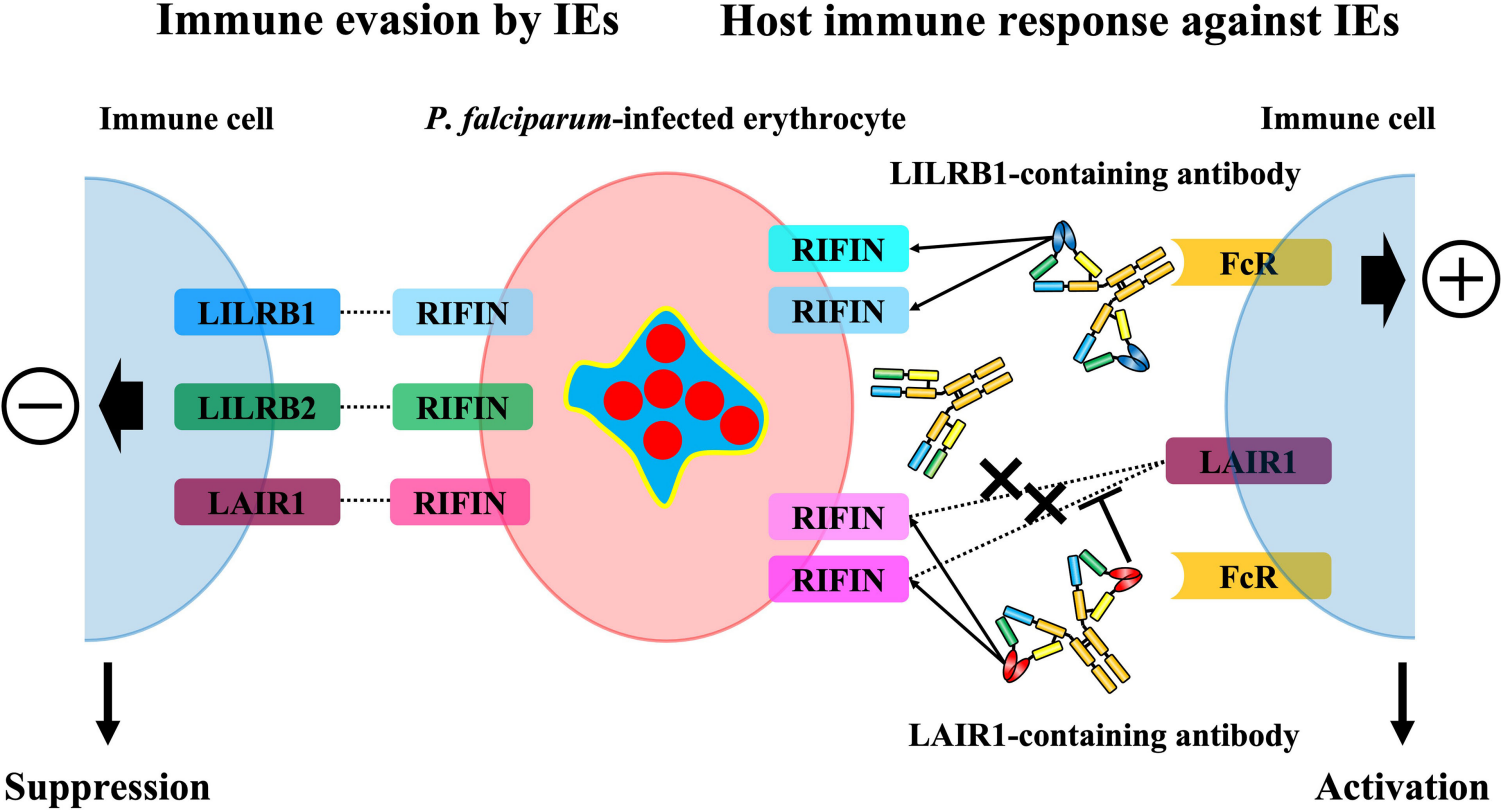
The immunosuppressive functions of RIFINs towards host immunity and the host immune response against RIFINs. Some RIFINs bind to the human inhibitory immune receptors and induce the inhibitory signal to the host immune system, and it is suggested that LILRB1-binding RIFINs may contribute to the severity of malaria. The host conversely induces normal antibodies against RIFINs and specialized antibodies that contain a portion of the immune receptors to block the RIFIN-receptor interaction and broadly recognize IEs expressing LAIR1 or LILRB1-binding RIFINs and promote the elimination of these IEs.

LILRB1 is an inhibitory receptor expressed on various immune cells, such as monocytes, T cells, B cells, and NK cells, that recognizes MHC class I molecules as physiological ligands to suppress immune responses to host cells and maintain host immune homeostasis (37). It has been reported that LILRB1 is exploited in viral host immune evasion mechanisms. UL18, an MHC class I like molecule derived from human cytomegalovirus (HCMV), is a well-studied molecule that binds to LILRB1 and suppresses immune cell responses (38, 39) (**Figure 2**). Dengue virus (DENV) also exploits LILRB1 to enhance the antibody-opsonized DENV infection into monocytes *via* the inhibition of Fc-gamma receptor (FcγR) signaling for type-I IFN-stimulated genes (ISGs) (40). Further, LILRB1 is known to contribute to the immune evasion of various cancers (**Figure 2**). LILRB1 blockade enhances anti-cancer immunity of NK cells and CD8^+^ T cells similar to the blockade of the PD-1/PD-L1 axis (25, 32, 41–44). The extracellular domain of LILRB1 is composed of 4 domains (D1-D4) and is reported to bind HLA class I molecules and other known ligands *via* D1D2 (45). Structural basis analysis revealed that LILRB1-binding RIFIN interacts with LILRB1 D1D2 through its variable region, mimicking the interaction between MHC class I molecules and LILRB1 (46). The function of LILRB1-binding RIFINs is to directly interact with LILRB1 on immune cells and induce the suppression of IgM production by B cells and the reduction of cytotoxicity of NK cells (23) (**Figure 3**). Furthermore, the analysis of infected erythrocytes from patients with malaria revealed that IEs in severe malaria cases expressed more LILRB1-binding RIFINs than did those in mild cases, suggesting that the RIFIN-LILRB1 interaction may contribute to the severity of the disease (23).

### -LILRB2-Binding RIFIN

LILRB2 is an inhibitory receptor of the LILR family expressed on myeloid immune cells such as monocytes, macrophages, and dendritic cells, and, similar to LILRB1, MHC class I molecules are its physiological ligands (47) (**Figure 2**). It has been reported that LILRB2 is involved in the host immune evasion mechanisms of human immunodeficiency virus (HIV) (48) and lung cancer (49) (**Figure 2**). For example, conventional dendritic cells (cDC) enhance the expression of LILRB2 and HLA class I molecules in the early stages of HIV infection, and this could be involved in early cDC dysfunction and the failure to induce subsequent adaptive immune responses to control HIV infection (50). The structure of LILRB2 is very similar to LILRB1, and its extracellular region has an 81% amino acid homology (45, 47). It has been reported that LILRB2 also binds to HLA class I molecules and other known ligands *via* D1D2 (47, 51, 52). However, LILRB2-binding RIFIN has been shown to interact with LILRB2 D3 through its variable region based on the results of a blocking assay with anti-LILRB2 antibodies and a binding assay with recombinant LILRB2 proteins (24) (**Figure 3**). This suggests a new binding mode of LILRB2 to the ligand through D3, and further studies are needed to clarify the function of this LILRB2-binding RIFIN.

### -LAIR1-Binding RIFIN

LAIR1 is an inhibitory receptor expressed on almost all immune cells and recognizes collagens (53) and molecules with collagen-like domains as physiological ligands (54, 55) (**Figure 2**). Overexpression of collagens by cancer cells is known to be associated with poor overall survival in several tumors such as lung (56, 57), colorectal (26, 58) and ovarian cancers (59, 60), and cancer cells are thought to exert LAIR-1-mediated immune evasion mechanisms through the remodeling of collagens in the tumor microenvironment (61) (**Figure 2**). Our study showed that some kinds of RIFINs interact with naïve LAIR1 (23), and recent conformational analysis revealed that LAIR1-binding RIFIN binds to LAIR1 *via* a variable region similar to LILRB1- and LILRB2-binding RIFINs (23, 24, 46). Furthermore, LAIR1-binding RIFINs are suggested to have immunosuppressive functions against LAIR1-expressing immune cells based on a LAIR1 reporter cell assay (62) (**Figure 3**).

### -Receptor-Containing Antibodies Targeting RIFINs

Receptor-containing antibodies with a portion of the LAIR1 and LILRB1 exons were produced in malaria patients in malaria-endemic areas (63, 64). These antibodies are types of broadly neutralizing antibodies (bnAbs) that can recognize multiple LAIR1- and LILRB1-binding RIFINs at once, and *in vitro* experiments have also shown ADCC activity of NK cells with LAIR1-containing antibodies (65) (**Figure 3**). Most of the identified LAIR1-containing antibodies have amino acid mutations in the collagen binding motifs of LAIR1, and these mutations reduce the binding affinity with collagen while increasing the binding affinity with RIFIN (66). Further, these LAIR1-containing antibodies block the interaction of LAIR1-binding RIFINs and LAIR1 on immune cells (62) (**Figure 3**). LILRB1-containing antibodies have LILRB1 D3D4 or D3 alone in the VH-CH1 elbow, and these antibodies bind to the variable region of LILRB1-binding RIFIN through its D3 (64), similar to the interaction of LILRB2 and LILRB2-binding RIFIN (24). These LILRB1-containing antibodies are unlikely to inhibit the same physiological function as LILRB1 because LILRB1 interacts with physiological ligands through its D1D2 (38). Therefore, receptor-containing antibodies can induce specific immune responses against *P. falciparum* without causing any autoimmune responses. This discovery is the first indication that the host uses unusual antibodies, such as receptor-containing antibodies, to induce immunity to *P. falciparum*.

## Conclusion

It has been seen that IE surface antigens possess mechanisms through which *P. falciparum* attempts to evade the host immune cell responses by interacting directly with host immune cells. Research on the interactions of human inhibitory immune receptors with RIFIN and other IE surface antigens is still at a preliminary stage. However, our understanding of ligand-receptor interactions not only reveals the overall immune evasion mechanisms used by *P. falciparum* but may also lead to the development of new malaria treatments and vaccines.

## Author Contributions

AS wrote the first draft of the manuscript. HA reviewed and commented on all drafts. All authors contributed to the article and approved the submitted version.

## Funding

The work was supported by JSPS KAKENHI grant number JP 18H05279 (HA) and MSD Life Science Foundation, Public Interest Incorporated Foundation (AS).

## Conflict of Interest

The authors declare that the research was conducted in the absence of any commercial or financial relationships that could be construed as a potential conflict of interest.

## Publisher’s Note

All claims expressed in this article are solely those of the authors and do not necessarily represent those of their affiliated organizations, or those of the publisher, the editors and the reviewers. Any product that may be evaluated in this article, or claim that may be made by its manufacturer, is not guaranteed or endorsed by the publisher.
